# Molecular Rearrangements in Protomembrane Models Probed by Laurdan Fluorescence

**DOI:** 10.3390/membranes13040386

**Published:** 2023-03-28

**Authors:** Loreto Misuraca, Roland Winter, Bruno Demé, Philippe M. Oger, Judith Peters

**Affiliations:** 1 Univ. Grenoble Alpes, CNRS, LIPhy, 38000 Grenoble, France; 2 Institut Laue Langevin, 38042 Grenoble, France; 3 Fakultät für Chemie und Chemische Biologie, Physikalische Chemie, Technische Universität Dortmund, 44227 Dortmund, Germany; 4 INSA Lyon, Université de Lyon, CNRS, UMR5240, 69100 Villeurbanne, France; 5 Institut Universitaire de France, 75005 Paris, France

**Keywords:** protomembranes, origin of life, thermal stability, small-angle neutron scattering, fluorescence with Laurdan

## Abstract

Lipid membranes are a key component of living systems and have been essential to the origin of life. One hypothesis for the origin of life assumes the existence of protomembranes with ancient lipids formed by Fischer–Tropsch synthesis. We determined the mesophase structure and fluidity of a prototypical decanoic (capric) acid-based system, a fatty acid with a chain length of 10 carbons, and a lipid system consisting of a 1:1 mixture of capric acid with a fatty alcohol of equal chain length (C10 mix). To shed light on the mesophase behavior and fluidity of these prebiotic model membranes, we employed Laurdan fluorescence spectroscopy, which reports on the lipid packing and fluidity of membranes, supplemented by small-angle neutron diffraction data. The data are compared with data of the corresponding phospholipid bilayer systems of the same chain length, 1,2-didecanoyl-*sn*-glycero-3-phosphocholine (DLPC). We demonstrate that the prebiotic model membranes capric acid and the C10 mix show formation of stable vesicular structures needed for cellular compartmentalization at low temperatures only, typically below 20 °C. They reveal the fluid-like lipid dynamic properties needed for optimal physiological function. High temperatures lead to the destabilization of the lipid vesicles and the formation of micellar structures.

## 1. Introduction

One hypothesis for the origin of life, which assumes the existence of protomembranes surrounding cells, is that ancient lipids were likely simple amphiphilic substrates as they could have been formed by Fischer–Tropsch processes [1,2]. One possible environment for the formation of such molecules was close to hot vents in the deep ocean, where sufficient energy was available and the ocean water protected biomolecular systems from the deleterious radiation effects of the young sun [3]. To investigate the dependence of such molecules on temperature and concentration covering the conditions in the deep sea, we carried out a systematic study employing Laurdan fluorescence spectroscopy on three samples: one containing only fatty acids, another one fatty acids in combination with fatty alcohols, and a third one that serves for comparison, containing modern phospholipids. The first one, decanoic (or capric) acid, is a fatty acid with a chain length of 10 carbons. The second sample corresponds to a possible architecture for protocell membranes, consisting of a 1:1 mixture of capric acid with a fatty alcohol of equal chain length (decanol). These short, single-chain amphiphilic molecules were readily available in the prebiotic environment and able to form stable vesicles [4,5]. In a recent paper, we described the remarkable properties of this membrane architecture, allowing to withstand extreme temperature conditions [6,7,8]. At room temperature, the combination of fatty acids and alcohols improves the vesicles’ stability, and it induces a high-temperature conformational change at *T* ≥ 60 °C that leads to vesicle fusion with stable membranes at temperatures as high as 80 °C. In the following, this model membrane will be referred to as C10 mix.

**Figure 1:**
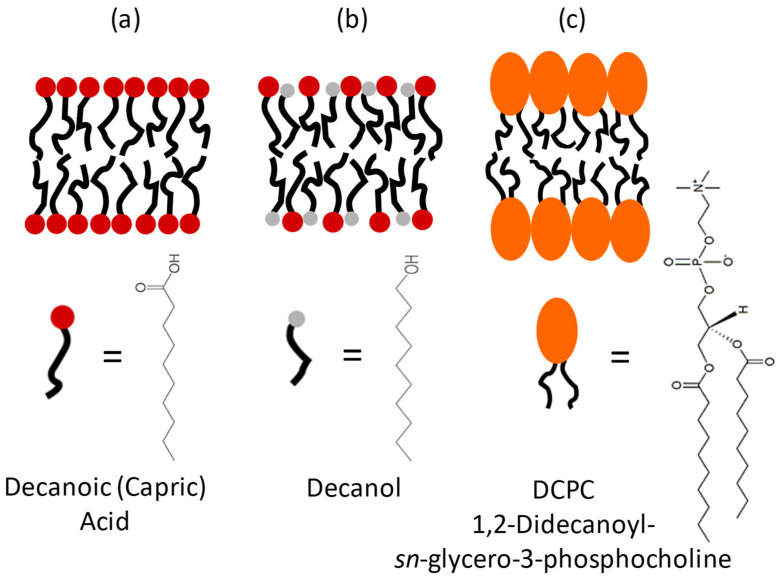
Three models for protomembranes: (**a**) Capric acid, (**b**) Capric acid + decanol (C10mix), (**c**) DCPC, and their chemical structures.

We compared the decanoic acid with a more evolved modern lipid, a phospholipid having two acyl chains with 10 carbons, but with the much bulkier choline headgroup: 1,2-didecanoyl-*sn*-glycero-3-phosphocholine (DCPC). The two acyl chains are bound to the headgroup through a glycerol backbone, which stiffens the structure (see Figure 1).

The fatty acids pass through a series of critical concentrations and temperatures that are mandatory to allow the formation of different phases. The Krafft temperature is the minimum temperature for surfactants to form micelles. Below the Krafft temperature, the critical micellar concentration (CMC) is not reached, and flocculation sets in, i.e., the colloidal particles sediment, forming flocs. DCPC does form micelles at very low concentrations, only [9]. The critical vesicle concentration (CVC) is defined as the minimum concentration needed to form vesicles. Fatty acids are known to have rather high CVCs, whereas fatty alcohols lower the CVC.

To shed light on the mesophase behavior of these prebiotic model membranes under various concentration and temperature conditions, we employed Laurdan fluorescence spectroscopy [10], which reports on the lipid packing and fluidity of the samples. Laurdan (6-dodecanoyl-N,N-dimethyl-2-naphthylamine) is a solvatochromic membrane fluorophore (with a chain length of 12 carbons) that partitions in the upper part of lipid bilayers. It is largely used to investigate lipid phase transitions, lipid packing, and hence membrane fluidity, as well as the hydration of lipid bilayer interfaces. The emission spectrum of Laurdan is centered at about 490 nm corresponding to lipids in a disordered phase and is shifted to blue (around 430 nm) when the lipids are in a more packed phase. The generalized polarization function, *GP* = (*I*_430_ − *I*_490_)/(*I*_430_ + *I*_490_), provides a suitable and quantitative way to measure the shift of the recorded emission intensities, I. Consequently, higher *GP* values indicate a higher lateral order, while decreasing *GP* values represent increasing disorder (fluidity) of the lipid acyl chains inside the membrane. Here, we are comparing the Laurdan fluorescence data with results from small-angle neutron scattering (SANS) data to be able to help identify the different phases encountered.

## 2. Materials and Methods

### 2.1. Sample Preparation

Decanoic acid, 1-decanol, DCPC, and bicine buffer were purchased from Sigma Aldrich (Merck, Rahway, NJ, USA). The samples were prepared by dissolving the decanoic acid and the decanol in a CHCl_3_ solution and DCPC in chloroform: methanol (2:1), followed by drying them under a stream of nitrogen. The samples were then placed in a desiccator and left under vacuum overnight. The sample weights were checked at each step, to make sure that all the organic solvent was evaporated. For the samples consisting of decanoic acid: 1-decanol (C10mix), the final ratio was 1:1.

The bicine buffer was prepared at a concentration of 0.2 M by dissolving hydrogenated bicine in H_2_O, following previous protocols [7]. The buffer was filtered with a 0.2 μm millipore membrane before use. The dried organic solutions were re-suspended in buffer by vigorous vortexing for ≈1 min, leading to the final milky-foamy solutions indicating the presence of large multi-lamellar vesicles (MLVs). The samples to be measured were then diluted in H_2_O buffer to their final concentration.

For the purpose of uniformity within the sample and to avoid light scattering, the samples were extruded with the help of the mini-extruder from Avanti Polar Lipids (Alabaster, AL, USA), using a 100 nm pore size polycarbonate membrane. The extrusions, undergoing 11 passes per sample, were performed with a heating block in thermal equilibrium at 40 °C and less than 24 h before each experiment.

For the study as a function of temperature, the concentrations were 80 mM for capric acid and the C10 mix and 40 mM for DCPC, so as to match the number of lipid tails. The dependence on concentration was investigated between 0.1 and 70 mM at 25 °C.

### 2.2. Fluorescence with Laurdan

The polycyclic aromatic compound naphthalene possesses a dipole moment that provokes the reorientation of water molecules resulting in a red shift of the probe emission [11]. After excitation at 350 nm, the emission spectrum of Laurdan between 390 and 550 nm in phospholipid membranes presents two local maxima, one at about 430 nm for ordered lipid (e.g., L_β′_) gel phases, which sometimes coexist with micelles as in capric acid, and the other around 490 nm for the more disordered and fluid (liquid-crystalline) lipid phases (L_α_). As the membrane is more disordered in the liquid-crystalline phase compared to the gel phase, the naphthalene moiety will be surrounded by more water molecules causing the red shift. To quantify the polarity change, the general polarization (*GP*) term is introduced using the emission intensities of the two local maxima which were here located at 430 and 490 nm, respectively [12]:

(1) $$GP=\frac{I\left(430\;\mathrm{nm}\right)-I\left(490\;\mathrm{nm}\right)}{I\left(430\;\mathrm{nm}\right)+I\left(490\;\mathrm{nm}\right)}$$

Laurdan *GP* values can range from +1 to −1, with −1 representing the highest lipid membrane fluidity [13]. *GP* values of around 0.5 denote the existence of ordered gel phases, whereas *GP* values close to and below zero are typical for all fluid-like lipid phases.

### 2.3. Small-Angle Neutron Scattering (SANS) Measurements

SANS is a scattering technique giving access to structural information at low resolution and permits to identify the mesophases of the MLVs studied here. For that, we measured DCPC and the C10mix with the D33 instrument [14] at the Institut Laue Langevin (ILL) in Grenoble, France, with three configurations combining different incident wavelengths *λ* (5 Å and 14 Å) and detector distances (2, 10, and 12 m). They correspond to a range of momentum transfers 0.001 Å^−1^ < *q* < 0.5 Å^−1^. The measurements were performed using a thermostated sample changer allowing access to a temperature range of 20 to 80 °C. More information about the technique and data analysis can be found in [7]. Capric acid was investigated on the high-resolution diffractometer D16 at the ILL [15] with neutron wavelength *λ* = 4.5 Å and a sample-detector distance of 0.95 m. The experiment was described in more detail in [6]. Shortly, the data were reduced by subtracting the signal from the sample container and the instrumental background. The quantity measured in a SANS experiment is the intensity *I*(*q*), where *q* is the momentum transfer between the neutron and the sample. It reads:

(2) $$I\left(q\right)=\left|F{\left(q\right)}^{2}\;S\left(q\right)\right|=\left|P\left(q\right)\;S\left(q\right)\right|$$

where *P*(*q*) is the particle form factor and *S*(*q*) the structure factor. In the present context, the particle form factor is of interest as it contains information about the shape of the scatterers, e.g., if they include vesicles or micelles and with which percentage. The fitting of the SANS curves was performed using the public code SASView (http://www.sasview.org/, accessed on 24 March 2023).

## 3. Results

### 3.1. Temperature Study

**Figure 2:**
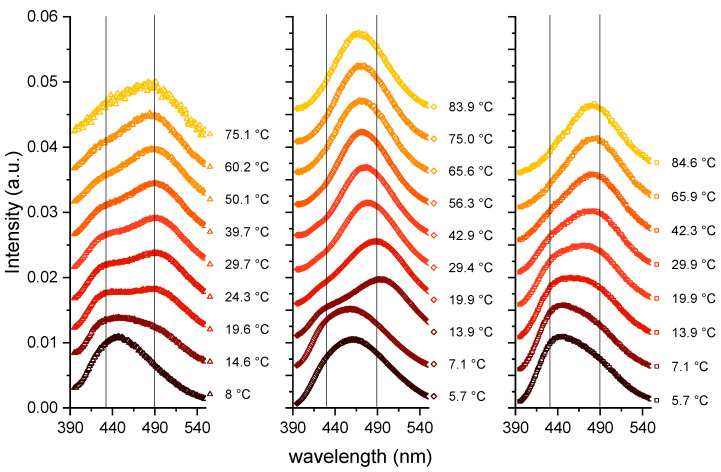
Laurdan spectra as a function of the emitted wavelength and as a function of temperature. Left: Capric acid; Middle: C10 mix; Right: DCPC. The vertical lines represent the wavelengths around 430 and 490 nm, which are characteristic of ordered and fluid lipid phases, respectively.

In the following, the concentrations used were 80 mM for capric acid and the C10 mix and 40 mM for DCPC to match the number of lipid tails. The first measurements were operated by increasing the temperature from about 6 to 85 °C (see Figure 2), and the Laurdan spectra are shown as a function of the emitted wavelength.

**Figure 3:**
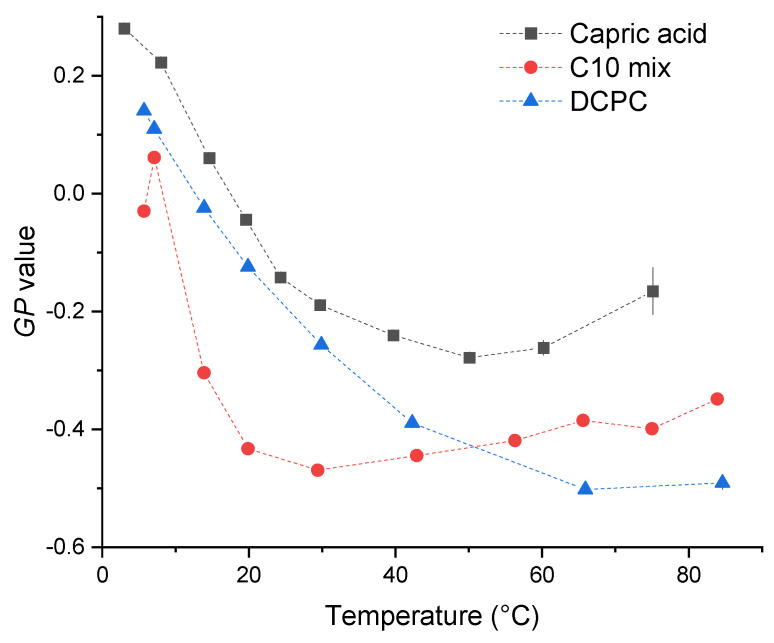
*GP* values calculated for the three samples as a function of *GP* temperature.

The three samples behave in a similar way: at the lowest temperature, they show the most ordered lipid conformation, indicated by a peak at around 430 nm. A certain number of kinks in the chains can be hypothesized, allowing some fluidity, but a fully ordered gel phase, consisting of trans-chain conformations only, is not reached as shown by the *GP* values calculated according to Equation (1) (see Figure 3), which are significantly below 0.5. When increasing the temperature, the maximum of the spectra is shifted to larger wavelengths, in a continuous way for DCPC, and to a value in between the two peaks for capric acid and the C10 mix. The *GP* value decreases continuously for DCPC and exhibits a shallow minimum for capric acid and the C10 mix at high temperatures.

The GP curve of DCPC follows the expected truncated sigmoidal shape [16]. In this temperature range, DCPC is always in a fluid-like phase, as the gel-to-fluid transition temperature is below 0 °C [17]. It is the most fluid lipid system among the three at very high temperatures, beyond about 60 °C. Conversely, the temperature dependence of the *GP* value of the other two samples is different, showing first a decrease, followed by a slight increase at high temperatures. Whereas the absolute minimum value becomes as low as for DCPC for the C10 mix sample, the *GP* values are always higher for capric acid, indicating a less fluid behavior.

**Figure 4:**
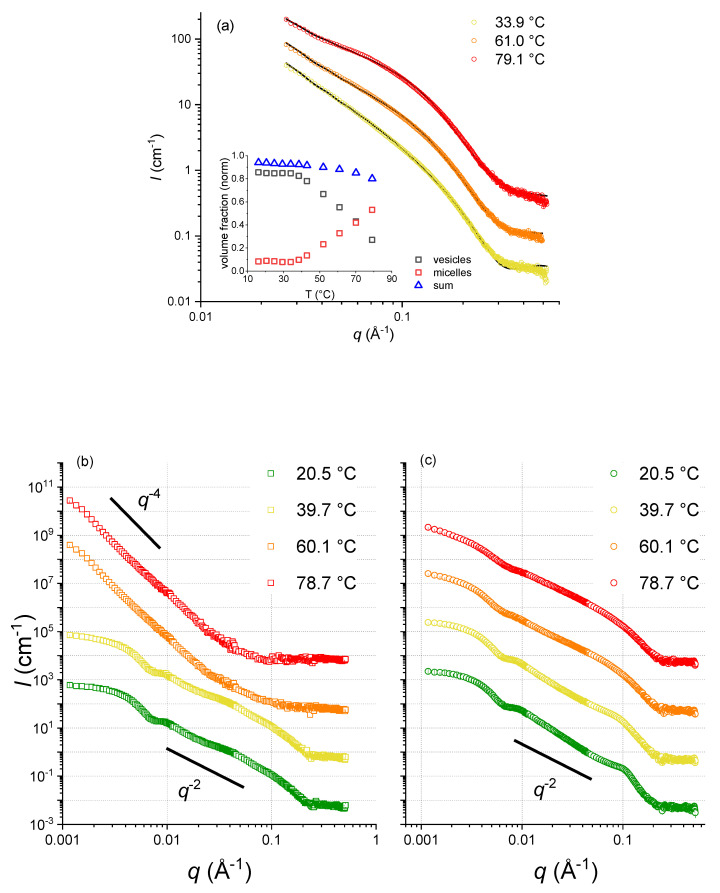
(**a**) SANS curves of capric acid samples (not extruded) for three temperatures. Inset: respective fraction of vesicles and micelles (reproduced and modified from [6]). (**b**) SANS curves of extruded C10 mix samples at increasing temperature. (**c**) SANS curves of extruded DCPC samples at the same increasing temperature ramp.

As Laurdan fluorescence cannot provide direct insights into structural information, we completed our studies with SANS experiments, carried out at the ILL. Although SANS is a low-resolution technique, it is very sensitive to the dimensions of micelles and vesicles (see Figure 4), allowing us to determine the mesophase structures of capric acid, the C10 mix [6], and DCPC. At lower temperatures, in the region 0.05 < *q* < 0.15 Å^−1^, the scattering intensity, I(q), follows a q^−2^ decay, which is compatible with lamellar form factors (vesicles formed by lipid bilayers). At higher temperature, the data show a significantly steeper decay, with a q^−4^ power law. This decay, also known as Porod law [18], is the signature of large aggregates modeled here with a spherical shape. According to these data, capric acid presents vesicles up to about 40 °C, where a dissolution of vesicles sets in, leading to the formation of spherical micelles, which are the dominant structural entities beyond about 70 °C (see the inset of Figure 4a). On the contrary, the C10 mix forms stable vesicles up to at least 60 °C. So, one could speculate that the phase behavior of capric acid could be due either to the intrinsic nature of the pure fatty acid membrane, not being “fluidified” by the alcohol insertion, or from a combination of effects due to vesicle—micelle coexistence and equilibrium shift [6]. Contrary to the two other samples, the scattering curves corresponding to DCPC were similar for the four temperatures, indicating no significant structural changes and a high stability with respect to temperature changes.

### 3.2. Concentration Dependent Measurements

**Figure 5:**
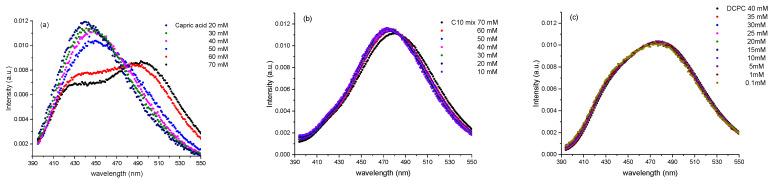
Laurdan emission spectra for (**a**) capric acid, (**b**) the C10 mix and (**c**) DCPC as a function of concentration at *T* = 25 °C.

A concentration series was performed at 25 °C for all samples in order to (1) find out which Laurdan fluorescence spectrum is representative of a micellar state, (2) to check whether micelles are present at a given concentration and in which samples, and (3) to make sure that there is no major modification to the signal due to the sample concentration (e.g., due to Rayleigh scattering). Figure 5 presents the emission spectra for the three samples as a function of concentration.

DCPC shows similar Laurdan emission spectral features with *GP* values around −0.2 over the whole concentration range, indicating that a vesicular mesophase structure is formed at all concentrations and that the system is in an overall fluid-like phase in accordance with what was found before. The C10 mix shows a similar behavior as DCPC with a very slight shift to higher wavelengths with increasing concentration, only. The *GP* value at 25 °C is around −0.5, which is again indicative of a pure fluid phase.

A surprising behavior is observed when performing dilution series of capric acid vesicles. In fact, at concentrations lower than the capric acid’s CVC (40 mM), the most intense peak visible is the one at 430 nm. This peak is due to pure Laurdan in bulk solution at low concentrations. Above the CVC, the Laurdan is incorporated into the lipid aggregate structure showing the expected broad peaks around 430 and 490 nm. The corresponding *GP* values are ~−0.2, indicating a fluid-like environment, as expected for a fluid vesicular/micellar system. Of note, micellar systems can adopt a fluid-like lipid state only, and cannot achieve a gel-like ordered chain packing.

In summary, DCPC and the C10mix are stable against concentration changes and present vesicles at all conditions with a peak close to 490 nm. Only capric acid undergoes a transition around 40 mM, the CVC of this type of lipids, where the peak is shifting from 430 nm to 490 nm when vesicles are formed.

## 4. Discussion and Conclusions

The methodological investigation presented here permits to assemble information from structural studies of the mesophases of all samples through SANS experiments and to combine them with Laurdan fluorescence spectra and parameters extracted from there. The following conclusions can be drawn.

All systems show the presence of vesicles with fluid-like lipid packing at low temperatures, for DCPC consisting of vesicles even up to temperatures as high as 80 °C, with multilamellar ones also at low temperatures. At high temperatures, the C10 mix undergoes a clearly visible structural change, as identified by the SANS curves showing a *q*^−4^ decay, which signifies the existence of spherical micellar structures, in agreement with the slight increase of the *GP* values beyond about 40 °C, which indicates a minor change in lipid packing. A similar behavior is observed for the capric acid with GP values larger than those of the C10 mix. This indicates a fluidizing effect imposed by the addition of the decanol to the fatty acid membrane, not only in the vesicular structures, but also in the micellar structures at high temperatures.

By contrast, typical phospholipids like DCPC with two lipid tails attached to the lipid headgroup do not undergo such transition as observed for the C10 mix. Indeed, the presence of two peaks in the Laurdan spectra at 430 and 490 nm (whose intensity increases with increasing temperature), respectively, and the q^−2^ decay of DCPC is preserved up to the highest temperature. The only difference at high temperatures is a progressive loss of the Guinier plateau in the neutron scattering data, suggesting that the vesicles are growing, fusing, and presenting a higher degree of polydispersity.

The *GP* values of all samples at the lowest temperature (around 5 °C) indicate a similar lipid packing, which is far less efficient than in a corresponding gel phase adopted by phospholipid systems of larger chain length, such as of di-C16 1,2-,dipalmitoyl-*sn*-glycero-3-phosphatidylcholine (DPPC), which shows a gel phase with *GP* values of ∼0.5 below the gel-to-fluid phase transition temperature of 41.5 °C. Stable vesicles of the prebiotic model membranes capric acid and the C10 mix show formation of stable vesicular structures needed for cellular compartmentalization at low temperatures, only, typically below 20 °C. They reveal the fluid-like lipid dynamic properties needed for optimal physiological function.

In combination with previous results on C10 carbon chain-based protomembranes, the current study confirms that a very stable membrane bilayer can be built from these simple lipids that were readily available on Earth at the origins of life. All studies point out the crucial role of fatty alcohols in improving membrane properties, which could have been the key to the origin of the first membranes. One has to remember that our study and previous ones were performed on mixtures of fatty acids and alcohols of the same chain length, which is somewhat unrealistic in nature. However, our results also suggest that the Incorporation of alcohols and acids of chain length smaller than 10 carbons, or longer, might further improve protomembranes’ properties. This hypothesis remains to be investigated experimentally.

## Data Availability

The raw neutron data is available upon request.
